# Explorative study of serum biomarkers of liver failure after liver resection

**DOI:** 10.1038/s41598-020-66947-1

**Published:** 2020-06-19

**Authors:** Kyung Chul Yoon, Hyung Do Kwon, Hye-Sung Jo, Yoon Young Choi, Jin-I Seok, Yujin Kang, Do Yup Lee, Dong-Sik Kim

**Affiliations:** 1 Department of Surgery, Division of HBP Surgery and Liver Transplantation, Korea University Medical Center, Korea University Medical College, Seoul, Korea; 2 Department of Bio and Fermentation Convergence Technology, BK21 PLUS Program, Kookmin University, Seoul, Korea; 3 Department of Agricultural Biotechnology, Center for Food and Bioconvergence, Research Institute for Agricultural and Life Sciences, Seoul National University, Seoul, Korea

**Keywords:** Hepatology, Liver

## Abstract

Conventional biochemical markers have limited usefulness in the prediction of early liver dysfunction. We, therefore, tried to find more useful liver failure biomarkers after liver resection that are highly sensitive to internal and external challenges in the biological system with a focus on liver metabolites. Twenty pigs were divided into the following 3 groups: sham operation group (n = 6), 70% hepatectomy group (n = 7) as a safety margin of resection model, and 90% hepatectomy group (n = 7) as a liver failure model. Blood sampling was performed preoperatively and at 1, 6, 14, 30, 38, and 48 hours after surgery, and 129 primary metabolites were profiled. Orthogonal projection to latent structures-discriminant analysis revealed that, unlike in the 70% hepatectomy and sham operation groups, central carbon metabolism was the most significant factor in the 90% hepatectomy group. Binary logistic regression analysis was used to develop a predictive model for mortality risk following hepatectomy. The recommended variables were malic acid, methionine, tryptophan, glucose, and γ-aminobutyric acid. Area under the curve of the linear combination of five metabolites was 0.993 (95% confidence interval: 0.927–1.000, sensitivity: 100.0, specificity: 94.87). We proposed robust biomarker panels that can accurately predict mortality risk associated with hepatectomy.

## Introduction

Liver resection is inevitable in the treatment of specific liver diseases. However, reduced liver volume can have many clinical manifestations such as small-for-size syndrome and post-hepatectomy liver failure, especially in cases of large volume resections of the liver. One of the main reasons for deteriorating liver function is high portal pressure, which results in high shear stress and sinusoidal injury^1^.

Conventional biochemical parameters for the evaluation of liver function in clinical settings, such as total bilirubin and prothrombin time (PT), are used as predictive markers. However, these biomarkers have limited usefulness in the prediction of early dysfunction. Total bilirubin and PT only have a 59% sensitivity in the prediction of postoperative mortality^2^. Therefore, there is a need for more specific and accurate predictive markers. Metabolites are the final readouts of a range of endogenous biochemical activities. Changes in the metabolic profile are highly sensitive to internal and external challenges in biological systems. Thus, comprehensive metabolome information has great value in the diagnosis and prognosis of various diseases.

In this study, we used liver failure animal models to determine changes in the levels of serum metabolites that can be identified before conventional biochemical markers. Two pig models were used to investigate the regenerating or failing liver after liver resection. Resection of 70% of the liver provided a model of liver injuries that did not lead to liver failure and served as a successful regenerative model, and resection of 90% of the liver provided a model of liver failure that eventually led to death. We identified key metabolites associated with specific metabolic consequences according to the remnant liver volume after hepatectomy including a range of metabolites associated with central carbon-nitrogen metabolism such as intermediates of glycolysis, the tri-carboxylic acid (TCA) cycle, amino acid metabolism, and fatty acid metabolism. The systematic integration of multivariate statistics revealed a unique metabolic feature according to the remnant liver volume after liver resection, and a predictive model for mortality following hepatectomy was constructed.

## Results

### Conventional biochemical analysis

**Figure 1:**
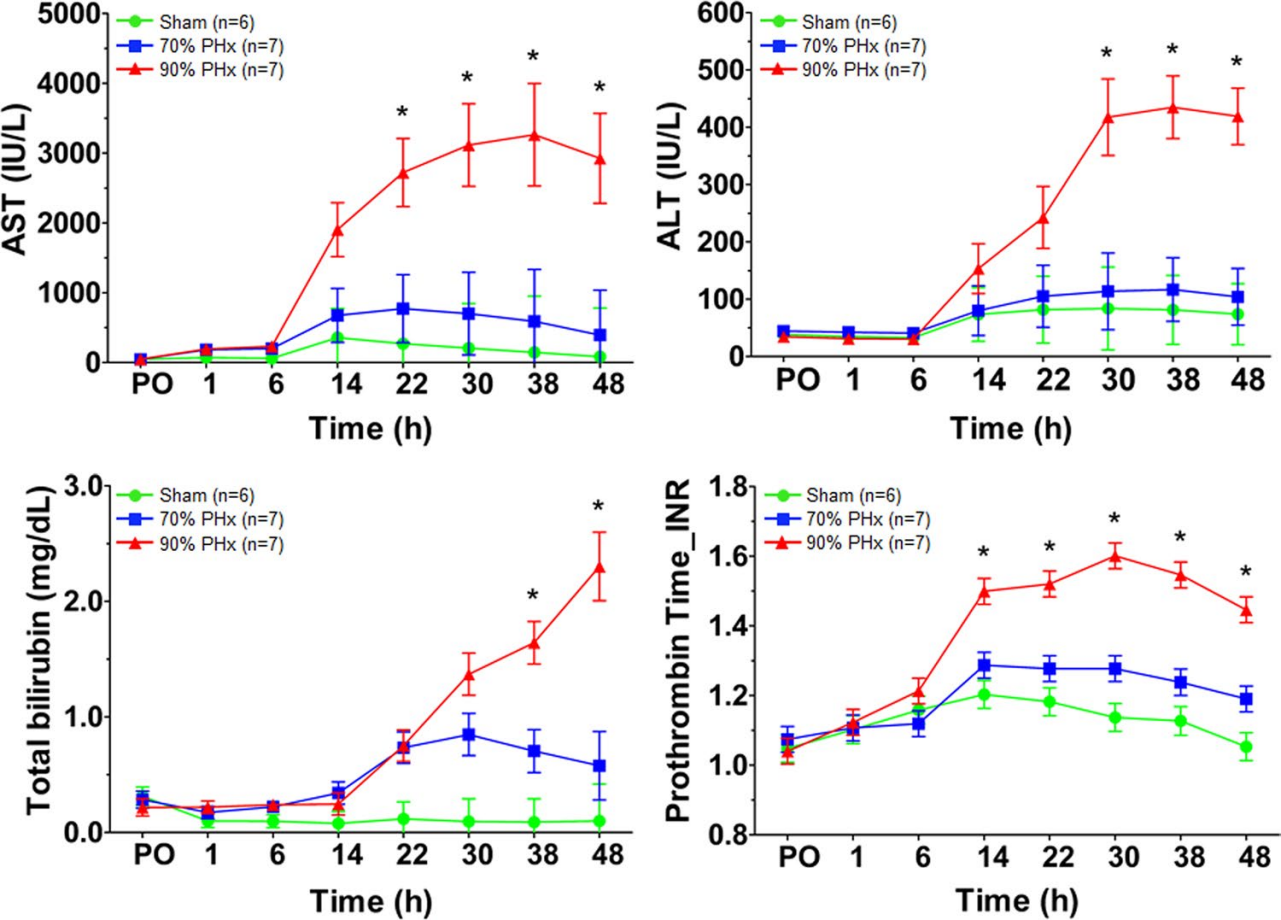
Serial changes of conventional biochemical markers (*P = 0.05, 70% *vs*. 90% liver resection, linear mixed model).

Figure 1 shows the serial changes in levels of total bilirubin (in mg/dL), aspartate aminotransferase (AST, in IU/L), and alanine aminotransferase (ALT, in IU/L) and in PT (INR). Total bilirubin level was significantly higher in the 90% resection group at 38 and 48 hours after surgery (*P* = 0.0014 and *P* = 0.0002, respectively), and AST level was higher at 22, 30, 38, and 48 hours after surgery (*P* = 0.015, *P* = 0.0128, *P* = 0.0314, and *P* = 0.0171, respectively). ALT level was significantly higher in the 90% resection group at 1, 30, 38 and 48 hours after surgery (*P* = 0.0453, *P* = 0.0047, *P* = 0.0002, and *P* < 0.0001, respectively). PT was significantly higher in the 90% resection group at 14, 22, 30, 38, and 48 hours after surgery (*P* = 0.0035, *P* = 0.0017, *P* = 0.0001, *P* = 0.0002, and *P* < 0.0001, respectively).

### Distinctive metabolic phenotypes according to mortality following hepatectomy

**Figure 2:**
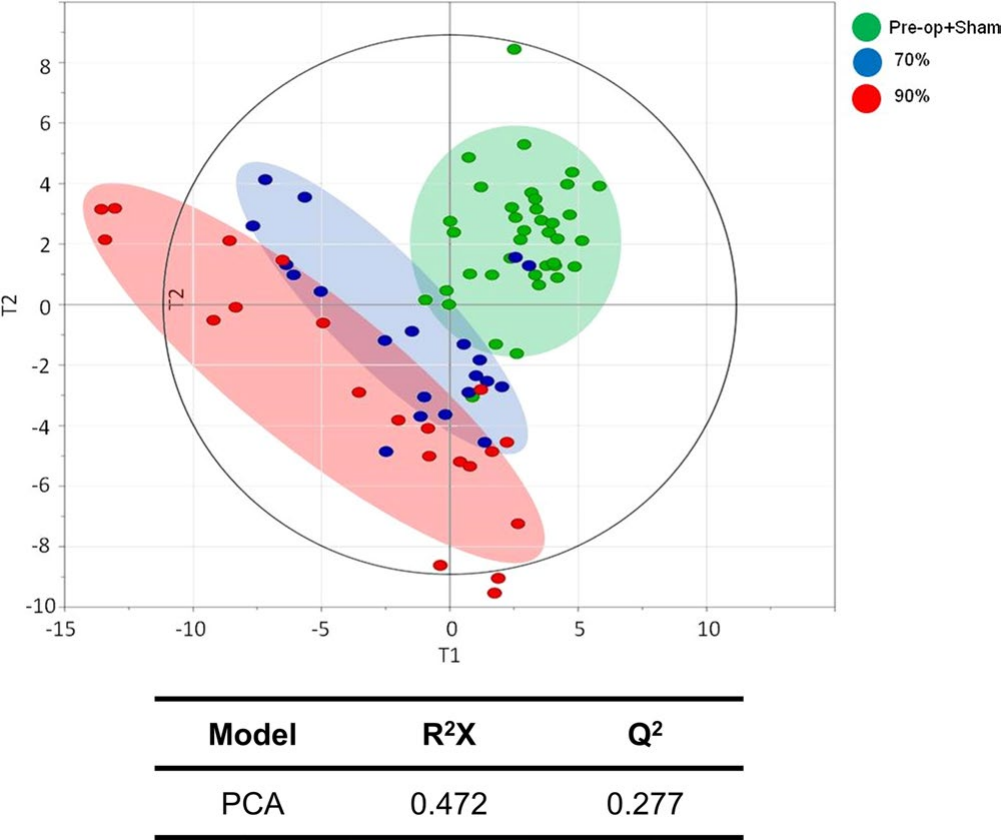
Unsupervised multivariate analysis based on principal component analysis. The resultant score plot shows the following 3 distinctive clusters: all samples in the preoperative stage and for the 70% and 90% hepatectomy groups following partial hepatectomy.

Untargeted metabolite profiling based on gas chromatography time-of-flight mass spectrometry (GC-TOF MS) revealed 129 compounds that fairly covered the primary metabolic pathways such as glycolysis, TCA cycle, amino acid metabolism, and fatty acid metabolism. Unsupervised multivariate statistics based on principal component analysis (PCA) revealed three major clusters. The profiles of the 90% hepatectomy group were clearly separated from the pre-operation and sham operation subjects whereas profiles of the 70% hepatectomy group were relatively similar to those of the pre-operation and sham operation groups (Fig. 2).

For further statistical analysis, data were adjusted for preoperative metabolite levels. After the adjustment, significant differences were observed in 48 compounds in the 90% hepatectomy group at 14 hours, 24 compounds at 30 hours, and 46 compounds at 48 hours, respectively (Supplementary Table 1). Significant differences were also observed between the 70% hepatectomy group and the sham operation group 27, 5, and 34 metabolites at 14, 30, and 48 hours, respectively.

**Figure 3:**
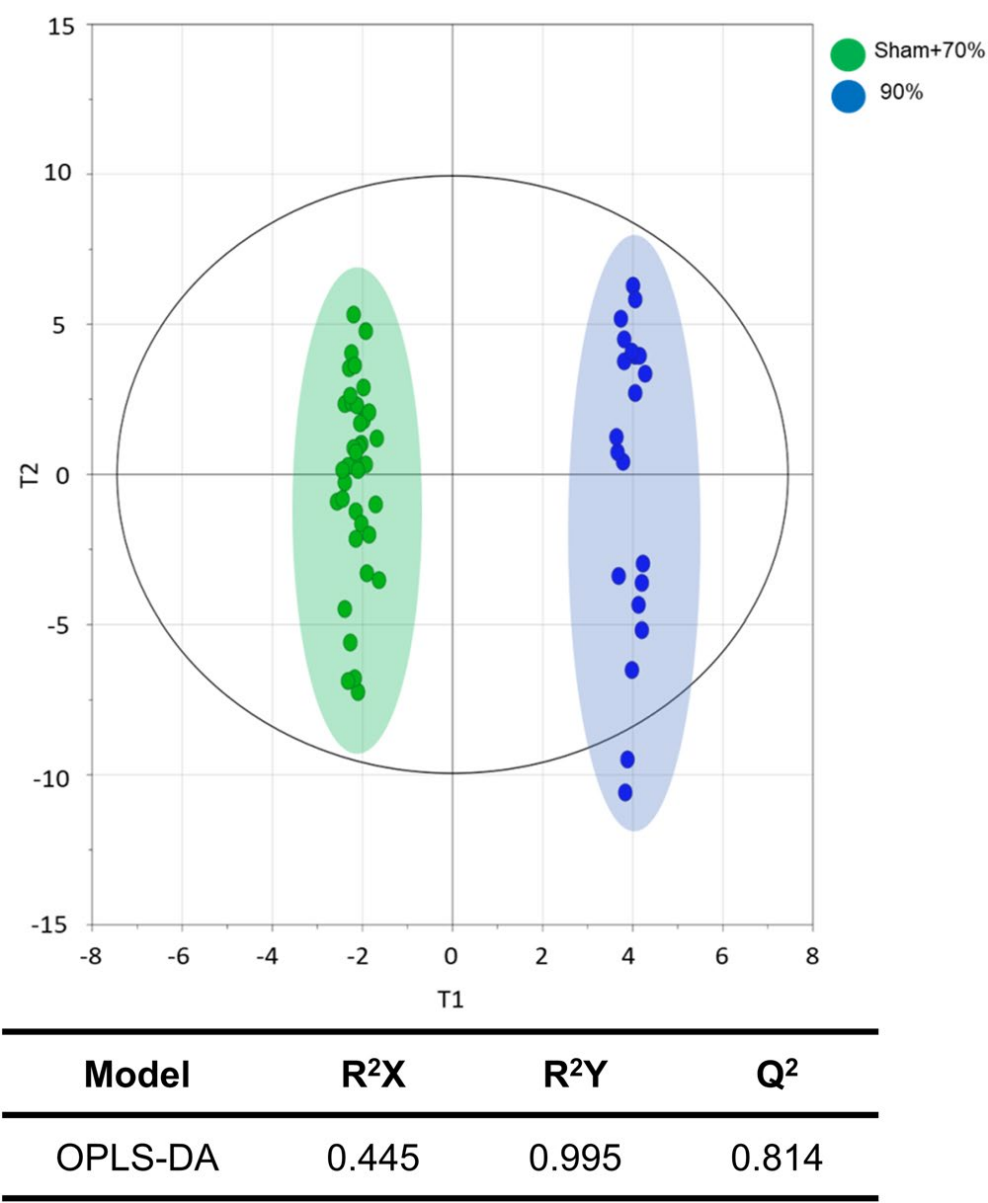
Discriminant model for hepatectomy-associated mortality (90% hepatectomy group) based on orthogonal projection in latent structures-discriminant analysis (OPLS-DA). Multivariate statistical model by OPLS-DA showing the highest level of an explained variance and predictability in the 90% hepatectomy group in contrast to the survival groups, the sham operation and 70% hepatectomy groups.

We used multivariate statistical models to systematically characterise the metabolic profiles unique to mortality after hepatectomy (90% hepatectomy group). We constructed a discriminant model for the 90% hepatectomy group that was distinct from the survival groups (i.e., the sham operation and 70% hepatectomy groups) at all time points. Based on orthogonal projection to latent structures-discriminant analysis (OPLS-DA) with 7-fold cross validation, high levels of explained variance and predictability were obtained (Q^2^Y of 0.995 and Q^2^ of 0.814) (Fig. 3). The variable importance in projection (VIP) analysis prioritised variables that contributed most to the discrimination (Fig. S1). The highest VIP score was observed for malic acid, followed by citric acid, xylitol, fumaric acid, phosphate, and tocopherol alpha. Subsequent pathway analysis of the metabolites (21 metabolites with VIP score > 1.2) showed that Ala-Asp-Glu metabolism, Arg-Pro metabolism, and TCA cycle were most significantly related to metabolic dysregulation in the 90% hepatectomy group (Fig. S2).

### Biomarker discovery

**Figure 4:**
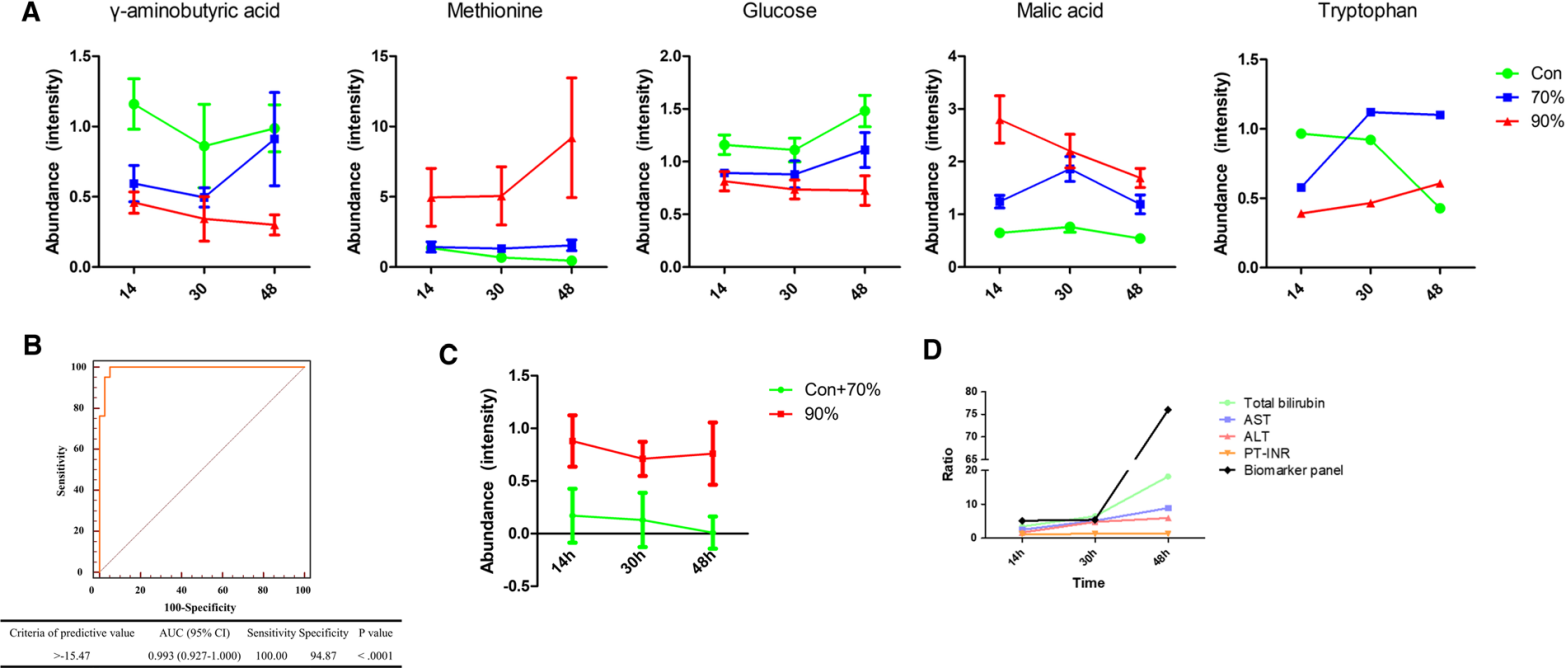
Performance evaluation of the biomarker panel (**A**) Line plots and statistical significance of the metabolites at each time point. Error bar indicates error of mean. (**B**) Receiver operating characteristic (ROC) curve analysis of serum metabolic biomarkers. The binary logistic regression model with malic acid, methionine, tryptophan, glucose, and γ-aminobutyric acid predicts the risk of hepatectomy lethality (90% hepatectomy from sham and 70% hepatectomy). (**C**) Line plots and statistical significance of the linearly transformed biomarkers. Error bar indicates error of mean. (**D**)The comparison of time profiles of the biomarker model and clinical markers. Y-axis is the ratio in which the values of 90% hepatectomy groups were divided by control groups (sham and 70% hepatectomy group).

To determine robust biomarkers that can predict the final outcome (survival or mortality), we conducted binary logistic regression analysis of the 21 metabolites. Multivariate analysis suggested five models that showed statistical significance for all selected variables. The recommended variables were levels of malic acid, methionine, tryptophan, glucose, and γ-aminobutyric acid (GABA) (Fig. 4A). The stepwise receiver operating characteristic (ROC) curve analysis showed good performance of the predictive models (Fig. S4). The area under the curve (AUC) of the linear combination of five metabolites was 0.993 (95% confidence interval: 0.927–1.000, sensitivity: 100.0, specificity: 94.87) (Fig. 4B). The risk of mortality following hepatectomy was determined using the formula: −12.95*(Glucose) + 5.93*(Malic acid) + 2.46*(Methionine) − 7.57*(Tryptophan) − 11.93*(GABA) − 7.34. We also tested the predictability of mortality at each time point. In contrast to the survival groups, the biomarker recomposite showed consistent discriminant power for the 90% hepatectomy group over all stages following hepatectomy. The AUCs were 0.989, 0.978, and 1.000 at 14, 30, and 48 hours, respectively. Likewise, the temporal profiles of the linear recomposite showed significantly higher levels in the 90% hepatectomy group for all time points (p < 0.001) (Fig. 4C). Further, we compared the predictive power of the biomarker panel to that of bilirubin, which is a conventional biochemical marker. ROC curve analysis showed the superiority of the metabolite biomarker signature, particularly at the early time point of 14 hours (Fig. S4). Moreover, the ratio (90% hepatectomy group over the survival groups) showed more dramatic differences by the biomarker panel relative to conventional markers during all time points (Fig. 4D).

## Discussion

The finding of an early biomarker means that liver failure prediction can be made, and the biomarker can be used to show the effects of management. Although there have been reports of positive effects of portal inflow modulation such as the use of a somatostatin analogue or terlipressin and splenectomy after major liver resection, there is, to date, no definite treatment for liver failure besides liver transplantation^3–5^. The purpose of this study was to find liver failure biomarkers that can be identified earlier than the conventional liver biomarkers.

This study is the first to investigate metabolomic characterisation for mortality prediction following hepatectomy using a pig model. Untargeted metabolic profiling systematically characterised metabolomic differences in blood serum according to remnant liver volume after resection. Further, we constructed a biomarker model of five serum metabolites that discriminated mortality after hepatectomy with high sensitivity and specificity over all time points under investigation.

The mechanism underlying liver failure after liver resection and dysfunction of small-size grafts is related to shear stress and sinusoidal injury^1^. Although efforts have been made to lower portal pressure and shear stress^1,6–9^, reports on biomarkers related to liver failure and dysfunction are currently inadequate. In clinical settings, the conventional markers of liver function include serum bilirubin, PT, AST, and ALT. AST and ALT are indicators of past injury, not indicators of present function. Even the combination of PT and total bilirubin, which are known as the most reliable predictive conventional markers, only has sensitivities of 14% and 19% on postoperative days 1 and 3, respectively^2^. The sensitivity increases to 59% on postoperative day 5. Additionally, clinical manifestations, such as ascites and neurological status, vary in the early postoperative period. Postoperative delirium, anaesthesia, and analgesia can affect neurological status, and intraoperative lymph node dissection and systemic volume status can affect the amount of ascites. Therefore, new biomarkers are needed for early detection, and we focused on liver metabolomes in this study^10^. To determine early biomarkers of liver failure, we used 70% and 90% liver resection models. A 70% liver resection is within the limits of safe resection, while a 90% resection is associated with liver failure leading to death^11,12^. To determine the biomarkers with the capability to discriminate patients with failing livers and exclude those with liver regeneration and in recovery after resection, we compared the 70% resection model with the liver failure model. Further, we sought to determine biomarkers that do not discriminate between the sham operation and 70% resection models. PT and ALT, which are conventional markers, were discriminative after 14 postoperative hours, while total bilirubin and AST did not distinguish between the 70% and 90% resection groups at every time point. This pattern is consistent with that reported in another study, which showed that PT was the only discriminative parameter with a value of approximately 1.5 (INR) in the 90% liver resection model^13^. PT has also been used to predict liver failure in clinical settings. However, PT alone is not enough for liver failure prediction. Bilirubin level on postoperative day 5 can be used in conjunction with PT to better predict liver failure; however, this only increases the prediction sensitivity to 59%^2^.

Based on untargeted metabolic profiling using GC-TOF MS, we observed that the primary metabolic physiology was clearly distinguishable according to the different volumes of remnant liver after hepatectomy. The profiles of all three groups prior to open surgery were analogous to that of the sham operation group postoperatively. Additionally, the 90% hepatectomy group was clearly distinguished from the other groups, whereas the 70% hepatectomy and sham groups had similar profiles. This suggests that open surgery alone has minimal effect on the blood metabolic profiles, and primary metabolic physiology can clearly distinguish between the different remnant liver volumes after hepatectomy.

The metabolic physiology in the 90% hepatectomy group was best characterised by differential regulation in bioenergetics and amino acid metabolism that was systematically identified using an integrative multivariate statistical model. A previous study that used nuclear magnetic resonance spectroscopy reported that energy metabolism was significantly downregulated by partial hepatectomy^14^, and this is consistent with our findings. The levels of two glycolysis intermediates, glucose and fructose-6-phosphate were lower in the 90% hepatectomy group than in the survival groups. In contrast, lactic acid level was significantly higher in the 90% hepatectomy group than in the survival groups, and it has been proposed that this is associated with acute and chronic hepatic insufficiency in patients^15^. There was also significant alteration of the TCA cycle, and this resulted in aberrant levels of citric acid, fumaric acid, and malic acid. It was recently reported that a significant decline in metabolite levels was found in the first half of the TCA cycle^16^. The OPLS-DA model revealed that the metabolites contributed most to the metabolic features of the 90% hepatectomy group.

We performed a binary logistic regression, which showed that the linear combination of five metabolites (malic acid, methionine, tryptophan, glucose, and GABA) sensitively predicted mortality following hepatectomy. The model discriminated the 90% hepatectomy group from the survival groups at all time points under investigation. GABA level gradually decreased and was lowest at the last time point in the 90% hepatectomy group (*p* > 0.08). In our study, we found that GABA level had a pattern that is opposite to that in previous studies that reported an inhibitory effect on hepatic regeneration following partial hepatectomy in rat models^17,18^. However, it has also been reported that GABA plays a protective role in acute liver injury^19^ and hepatotoxicity^20^. The metabolites need to be considered to play pivotal roles determining the liver regenerative capacity following partial hepatectomy in future investigations. In contrast, the difference in methionine levels gradually increased with time in the 90% hepatectomy group. A previous study reported that methionine-enriched diets instigated liver injury in cystathione-β-synthase-deficient mice as the consequent accumulation of homocysteine impaired liver regeneration after partial hepatectomy in a rat model^21^. Increase in methionine levels was noted in cases of severe liver failure in clinical settings, but not in cases of chronic active hepatitis^22,23^. Serum methionine concentrations greater than 200 μmol/L were found only in patients with severe liver failure, but not in patients with compensated viral hepatitis^23^. Additionally, significantly higher methionine levels were observed concomitantly with signs of decompensation such as ascites, jaundice, and hepatic encephalopathy^22^. There are two possible mechanisms underlying high levels of serum methionine in a failing liver. One is the disruption of the methionine cycle and the other is compensatory reactions to protect from further liver damage. The methionine cycle is one of the vulnerable cycles in liver disease, and methionine level varies depending on liver function and degree of compensation. S-adenosylmethionine synthetase (SAMe) level is higher in the early stages of liver disease and leads to low methionine levels. However, SAMe level eventually decreases during the compensatory periods, and this in turn causes an increase in methionine levels during the liver failing process^24^. With regard to compensatory reactions after massive surgery, the level of 5-methylthioadenosine (MTA), which is a downstream derivative of SAMe, increases via increase in the SAMe level. MTA has the capacity to protect the liver by increasing glutathione level, improving membrane stability, and decreasing the activity of TNFα^25,26^. Consequently, higher MTA levels result in higher methionine levels. In a previous study, this process was found to occur more actively in the acutely failing liver, especially in the group that received a somatostatin analogue with protective effect on the remnant liver after liver resection^27^. Both mechanisms may occur concomitantly 48 hours after massive surgery. The exact cut-off level is yet to be determined, and further serial examinations will help in the prediction of prognosis and liver failure in such patients.

The kynurenine pathway for tryptophan catabolism mainly occurs in the liver; however, tryptophan level varies depending on liver function and availability of the rate-limiting enzyme (indoleamine 2,3-dioxygenase) induced by inflammatory response and kidney function^28^. A recent prospective study revealed that, in the 70% resection model with compensated liver function, serum tryptophan level increased with normal or mildly elevated indoleamine 2,3-dioxygenase level^29^. Conversely, in the 90% resection model with decompensated liver function, tryptophan level decreased with increase in indoleamine 2,3-dioxygenase level induced by other organs such as the kidney^29^. Given the similar inflammatory states in the 70% and 90% resection models, it is unclear if compensation determines the tryptophan level^26,27,29^.

As described, high malic acid level is associated with TCA cycle disorders, and elevated levels of fumaric acid may lead to increased malic acid production. Urea cycle dysfunction is also associated with malic acid accumulation as aspartic acid consumption is reduced^30^. The level of malic acid, unlike that of other TCA metabolites, is affected by the urea cycle. The compensatory action of high malic acid level may be due to fumaric acid, which generates oxaloacetate and aspartic acid to promote the transaminase process. However, in patients with urea cycle disorders, this may lead to malic acid accumulation.

This study is meaningful as liver biomarkers in patients with acutely failing livers differ compared to other metabolites which are determined using untargeted metabolic profiling. In the clinical setting, it should be considered that these metabolic markers (malic acid and methionine) may vary during the failing process because of compensatory reactions. However, these markers can be monitored in patients with acute liver failure after hepatectomy at 48 postoperative hours. It should also be kept in mind that underlying diseases in a patient may affect the methionine cycle. In patients with hepatocellular carcinoma, the levels of glutamine, malic acid, and methionine may be elevated due to urea cycle disorders^30^. Furthermore, human hepatitis B-related liver cirrhosis leads to increase in urine and serum methionine levels^31^. We used a 90% hepatectomy model that leads to definite liver failure to find the biomarkers. However, there are limitations regarding determination of the exact cut-off level of the biomarkers in patients with marginal liver function in the clinical setting. Further study is needed to determine and validate cut-off levels of metabolomics biomarkers.

In summary, time-dependent profiling of serum metabolome characterized the metabolic features specific to mortality following hepatectomy. Primary dysregulation was identified in central carbon-nitrogen metabolism including glycolysis, TCA cycle, and amino acid metabolism. Systematic prioritization based on OPLS-DA and binary logistic regression analysis proposed robust biomarker panels that can accurately predict the risk of mortality associated with hepatectomy.

Nevertheless, our data has some limitations including sample size. Biomarker study based on high-throughput molecular profiling (e.g., metabolomics) is conducted in an untargeted and hypothesis-free way^32^. This often limits sample size determination for appropriate statistical power. According to a univariate statistic (e.g., analysis of variance [ANOVA]), to meet a false discovery rate (FDR) of 0.2, the sample size of our study should be 80 per group.

## Methods

### Animals and study design

We used 2–3-month-old domestic female crossbred Yorkshire-landrace and Duroc pigs weighing 35–40 kg. Twenty pigs were divided into the following 3 groups: sham operation group (n = 6), 70% hepatectomy group (n = 7), and 90% hepatectomy group (n = 7). The 70% and 90% hepatectomies were performed as described by Hori *et al*.^11^ The pigs were fasted for 8 hours before surgery and housed in individual pens maintained at 22 ± 1 °C in ambient humidity. The animals were sacrificed 48 hours after the hepatectomy.

### Liver resection and blood sampling

Liver resection was performed by board-certified hepato-biliary-pancreatic surgeons. Blood sampling was performed preoperatively and at 1, 6, 14, 30, 38, and 48 hours after surgery through a central venous catheter inserted via the right jugular vein before laparotomy. Serial measurements of blood AST, ALT, total bilirubin, and metabolite levels and PT were performed.

### Animal rights

This study was approved by the Korea University Institutional Animal Care and Use Committee (IRB: KOREA-2017–0069-C3), and all animals were cared for in accordance with the national guidelines for ethical animal research.

The research was also approved by the Governmental Committee on Animal Care; animals were cared for in compliance with institutional guidelines. Following the experimental protocol, the animals were sacrificed in deep anaesthesia using intravenous injection of potassium chloride (2 mmol/kg).

### Primary metabolite profiling by gas chromatography time-of-flight mass spectrometry

Metabolite extraction, derivatisation, and mass spectrometry analysis were conducted in a randomized order. Serum samples were thawed on ice at 4 °C, and 50 µL was mixed with 750 µL of extraction solvent (methanol:isopropanol:water, 3:3:2, v/v/v)^33^. Following 5 minutes of sonication and centrifugation, 700 µL of each supernatant was aliquoted into a new 1.5 mL tube. The aliquots were concentrated to complete dryness using a speed vacuum concentrator (SCANVAC, Korea). The dried extracts were then stored at −80 °C until derivatization.

The dried extracts were derivatized with 5 µL of 40 mg/mL methoxyamine hydrochloride (Sigma-Aldrich, St. Louis, MO, USA) in pyridine (Thermo, USA) and with 45 µL of N-methyl-N-trimethylsilyltrifluoroacetamide (MSTFA + 1% TMCS; Thermo, USA)^34^. A mixture of 13 fatty acid methyl esters was added as an internal retention index marker (C8, C9, C10, C12, C14, C16, C18, C20, C22, C24, C26, C28, and C30)^35^. Chromatographic separation was performed using Agilent 7890B gas chromatography (Agilent Technologies) equipped with an RTX-5Sil MS column (Restek, Gellefonte, PA, USA). Mass spectrometry analysis was performed on a LECO Pegasus HT time-of-flight mass spectrometer controlled by Chroma TOF software 4.50 version (LECO, St. Joseph, MI, USA)^36^.

### Data processing and statistical analysis

The conventional biochemical markers evaluated in clinical settings (total bilirubin, AST, ALT, and PT) were compared using the linear mixed model and ANOVA at each time point with the Tukey-Kramer test for the adjustment for multiple comparisons.

Mass spectrometric data pre-processing was conducted using Chroma TOF software upon data acquisition. Peak apex mass values, entire spectrum values, retention time, peak purity, and signal-to-noise ratio were acquired. Post-processing was performed based on the BinBase algorithm, which includes chromatogram validation, primary RI detection and calculation, and quant mass validation^37^. Quantitative values were calculated based on the peak height of a single quant mass. Missing values replacement was performed based on the BinBase algorithm, which processes raw data for post-matching and replacement^38^. For quality control purposes, every tenth sample of a mixture of 33 pure reference compounds was analyzed, and this ensured reproducible performance during the analysis^39^.

Metabolomic data were normalised based on the MSTUS method^40^ implemented in NOREVA (http://idrb.zju.edu.cn/noreva/)^41^. The normalised data were then adjusted according to the preoperative metabolite levels, and line plots were drawn for the sham operation group and the 70% and 90% hepatectomy groups. PCA, OPLS-DA, and VIP analysis were performed using SIMCA 15 (Umetrics AB, Umea, Sweden). The OPLS-DA model was validated based on 2-, 3-, 4-, and 7-fold cross validation. Univariate statistical analysis was performed using the Student’s t-test (EXCEL, Microsoft Office 365). Significance analysis of microarray (SAM) was applied to control the FDR. Binary logistic regression analysis (forward selection) was performed using IBM SPSS Statistics for Windows, version 25.0 (IBM Corp., Armonk, N.Y., USA). Kolmogorov-Smirnov and Shapiro-Wilk tests were conducted on the linearly transformed variables for normality check using IBM SPSS Statistics for Windows, version 25.0 (IBM Corp., Armonk, N.Y., USA). ROC analysis was performed using MedCalc for Windows, version 12.7.0.0 (MedCalc Software, Ostend, Belgium). Pathway over-representation analysis was performed based on the hypergeometric test and relative-betweenness centrality implemented in *MetaboAnalyst* (https://www.metaboanalyst.ca/).

## Supplementary information

Supplementary Information.

## References

[1] Xu X (2006). Attenuation of acute phase shear stress by somatostatin improves small-for-size liver graft survival. Liver Transpl.

[2] Balzan S (2005). The “50-50 criteria” on postoperative day 5: an accurate predictor of liver failure and death after hepatectomy. Ann Surg.

[3] Zhou L (2012). Serum metabolomics reveals the deregulation of fatty acids metabolism in hepatocellular carcinoma and chronic liver diseases. Anal Bioanal Chem.

[4] Hammond JS (2019). The effects of terlipressin and direct portacaval shunting on liver hemodynamics following 80% hepatectomy in the pig. Clin Sci (Lond).

[5] Takamatsu Y (2018). Intentional Modulation of Portal Venous Pressure by Splenectomy Saves the Patient with Liver Failure and Portal Hypertension After Major Hepatectomy: Is Delayed Splenectomy an Acceptable Therapeutic Option for Secondary Portal Hypertension?. Am J Case Rep.

[6] Umeda Y (2008). Effects of prophylactic splenic artery modulation on portal overperfusion and liver regeneration in small-for-size graft. Transplantation.

[7] Troisi R (2005). Effects of hemi-portocaval shunts for inflow modulation on the outcome of small-for-size grafts in living donor liver transplantation. Am J Transplant.

[8] Baik SK (2005). Acute hemodynamic effects of octreotide and terlipressin in patients with cirrhosis: a randomized comparison. Am J Gastroenterol.

[9] Fahrner R (2014). Elevated liver regeneration in response to pharmacological reduction of elevated portal venous pressure by terlipressin after partial hepatectomy. Transplantation.

[10] Yigitler C (2003). The small remnant liver after major liver resection: how common and how relevant?. Liver Transpl..

[11] Hori T (2014). How to successfully resect 70% of the liver in pigs to model an extended hepatectomy with an insufficient remnant or liver transplantation with a small-for-size graft. Surg. Today.

[12] Golriz M (2017). Establishing a Porcine Model of Small for Size Syndrome following Liver Resection. Can J Gastroenterol Hepatol.

[13] Court FG (2004). Subtotal hepatectomy: a porcine model for the study of liver regeneration. J Surg Res.

[14] Kooby DA (2000). Use of phosphorous-31 nuclear magnetic resonance spectroscopy to determine safe timing of chemotherapy after hepatic resection. Cancer Res..

[15] Heinig RE, Clarke EF, Waterhouse C (1979). Lactic acidosis and liver disease. Arch. Intern. Med..

[16] Saito Y (2018). Changes of liver metabolites following hepatectomy with ischemia reperfusion towards liver regeneration. Ann. Gastroenterol. Surg..

[17] Minuk GY, Gauthier T (1993). The effect of gamma-aminobutyric acid on hepatic regenerative activity following partial hepatectomy in rats. Gastroenterology.

[18] Wang S (2017). Protective roles of hepatic GABA signaling in acute liver injury of rats. Am. J. Physiol. Gastrointest. Liver Physiol..

[19] Wang S, Zhang L, Liu C, Lu WY (2017). Protective roles of hepatic GABA signaling in liver injury. Int. J. Physiol. Pathophysiol. Pharmacol..

[20] Norikura T, Kojima-Yuasa A, Opare Kennedy D, Matsui-Yuasa I (2007). Protective effect of gamma-aminobutyric acid (GABA) against cytotoxicity of ethanol in isolated rat hepatocytes involves modulations in cellular polyamine levels. Amino Acids.

[21] Liu WH (2010). Hepatocyte proliferation during liver regeneration is impaired in mice with methionine diet-induced hyperhomocysteinemia. Am. J. Pathol..

[22] Higashi T (1982). Impaired metabolism of methionine in severe liver diseases. I. Clinical and pathophysiological significance of elevated serum methionine levels. Gastroenterol Jpn.

[23] Kaldor J, Spelman DW, Metcalf WR, Lucas CR (1986). Serum and urinary methionine concentrations in severe hepatic failure of viral hepatitis. Relevance to development of encephalopathy and prognosis. Med J Aust.

[24] Kharbanda KK (2013). Methionine metabolic pathway in alcoholic liver injury. Curr Opin Clin Nutr Metab Care.

[25] Mato JM, Lu SC (2007). Role of S-adenosyl-L-methionine in liver health and injury. Hepatology.

[26] Anstee QM, Day CP (2012). S-adenosylmethionine (SAMe) therapy in liver disease: a review of current evidence and clinical utility. J Hepatol.

[27] Du Z (2016). Octreotide prevents liver failure through upregulating 5’-methylthioadenosine in extended hepatectomized rats. Liver Int.

[28] Saleem DM, Haider S, Khan MM, Shamsi T, Haleem DJ (2008). Role of tryptophan in the pathogenesis of hepatic encephalopathy. J Pak Med Assoc.

[29] Claria J (2019). Orchestration of Tryptophan-Kynurenine Pathway, Acute Decompensation, and Acute-on-Chronic Liver Failure in Cirrhosis. Hepatology.

[30] Yu M, Zhu Y, Cong Q, Wu C (2017). Metabonomics Research Progress on Liver Diseases. Can J Gastroenterol Hepatol.

[31] Chen T (2011). Serum and urine metabolite profiling reveals potential biomarkers of human hepatocellular carcinoma. Mol. Cell. Proteomics.

[32] Blaise BJ (2016). Power analysis and sample size determination in metabolic phenotyping. Analytical chemistry.

[33] Lee DY, Fiehn O (2008). High quality metabolomic data for Chlamydomonas reinhardtii. Plant Methods.

[34] Lee J-E, Cho YU, Kim KH, Lee DY (2016). Distinctive metabolomic responses of Chlamydomonas reinhardtii to the chemical elicitation by methyl jasmonate and salicylic acid. Process Biochemistry.

[35] Park SJ (2019). Integrative metabolomics reveals unique metabolic traits in Guillain-Barré Syndrome and its variants. Scientific reports.

[36] Lee EM (2019). Highly geographical specificity of metabolomic traits among Korean domestic soybeans (Glycine max). Food Research International.

[37] Polyzos, A. A., *et al*. Metabolic Reprogramming in Astrocytes Distinguishes Region-Specific Neuronal Susceptibility in Huntington Mice. *Cell metabolism* (2019). 10.1016/j.cmet.2019.03.004 PMC6583797 30930170

[38] Cho YU (2017). Exploratory metabolomics of biomarker identification for the internet gaming disorder in young Korean males. J. Chromatogr. B Analyt. Technol. Biomed. Life Sci..

[39] Ji DY (2018). Comparative assessment of Graves’ disease and main extrathyroidal manifestation, Graves’ ophthalmopathy, by non-targeted metabolite profiling of blood and orbital tissue. Scientific reports.

[40] Karpievitch YV, Nikolic SB, Wilson R, Sharman JE, Edwards LM (2014). Metabolomics data normalization with EigenMS. PLoS One.

[41] Moussaoui, D., *et al*. Early complications after liver transplantation in children and adults: Are split grafts equal to each other and equal to whole livers? *Pediatr Transplant* **21**(2017). 10.1111/petr.12908 28261944

