# Supplementary material for: Explorative study of serum biomarkers of liver failure after liver resection
Source: Sci Rep. 2020 Jun 19;10:9960. doi: 10.1038/s41598-020-66947-1 (PMC7305107; doi:10.1038/s41598-020-66947-1)
Supplement: Supplementary file 1 — Supplementary Information. [file 41598_2020_66947_MOESM1_ESM.docx]

<Supplementary file>

**Title: Explorative study of serum biomarkers of liver failure after liver resection**

Authors

Kyung Chul Yoon^1^, Hyung Do Kwon^2^, Hye-Sung Jo^1,^, Yoon Young Choi^1^, Jin-I Seok^1^, Yujin Kang^3^, Do Yup Lee^3*^ & Dong-Sik Kim^1*^

^1^Department of Surgery, Division of HBP Surgery and Liver Transplantation, Korea University Medical Center, Korea University Medical College, Seoul, Korea.

^2^Department of Bio and Fermentation Convergence Technology, BK21 PLUS Program, Kookmin University, Seoul, Korea

^3^Department of Agricultural Biotechnology, Center for Food and Bioconvergence, Research Institute for Agricultural and Life Sciences, Seoul National University, Seoul, Korea

^*^Corresponding authors:

Do Yup Lee, PhD., Department of Agricultural Biotechnology, Center for Food and Bioconvergence, Research Institute for Agricultural and Life Sciences, Seoul National University, Seoul, Korea, Seoul 08826, Korea; Tel: +82-10-9918-9338; Fax+82-2-873-5095; e-mail: [rome73@snu.ac.kr](mailto:rome73@snu.ac.kr)

Dong-Sik Kim, MD, PhD., Department of Surgery, Division of HBP Surgery and Liver Transplantation, Korea University Medical Center, Korea University College of Medicine, Seoul 02841, Korea. Tel: +82-2-920-6620; Fax: +82-2-921-6620; e-mail: [kimds1@korea.ac.kr](mailto:kimds1@korea.ac.kr)

Kyung Chul Yoon and Hyung Do Kwon contributed equally to this work.

**Conflict of interest statement**

The authors declare that there is no conflict of interest regarding the publication of this article

**Financial support statement**

This research was supported by the Bio & Medical Technology Development Program of the National Research Foundation (NRF) funded by the Ministry of Science and ICT of Korea (2018M3A9F3020970).

Table S1. The number of metabolites that were significantly changed in the hepatectomy-associated mortality group (90% liver resection group) compared to the survival groups (sham and 70% hepatectomy group)

| Time | Compounds | Up-regulation | Down-regulation |
| --- | --- | --- | --- |
| 14h | 48 | 25 | 23 |
| 30h | 24 | 13 | 11 |
| 48h | 46 | 12 | 34 |

Fig. S1. The list of metabolites with high variable importance in projection (VIP) scores based on the OPLS-DA model (VIP score > 1.2)

| Compounds | VIP Score |
| --- | --- |
| Malic acid | 1.80 |
| Citric acid | 1.73 |
| Xylitol | 1.70 |
| Fumaric acid | 1.67 |
| Phosphate | 1.62 |
| Tocopherol alpha | 1.50 |
| Threonic acid | 1.49 |
| Serine | 1.47 |
| Cholesterol | 1.43 |
| Lactic acid | 1.43 |
| Methionine | 1.41 |
| Thymine | 1.38 |
| β-alanine | 1.35 |
| Glucose | 1.35 |
| Ribitol | 1.34 |
| γ-aminobutyric acid | 1.33 |
| Anthosine | 1.33 |
| Tryptophan | 1.31 |
| Oxalic acid | 1.30 |
| Xanthine | 1.28 |
| Glutamate | 1.23 |

Fig. S2. Pathway over-representation analysis of the metabolites with VIP score > 1.2. Pathway impact value and statistical significance (-log(P) are represented on the X-axis and Y-axis, respectively. Likewise, the node color and size are determined by the –log(P) pathway impact value (relative-betweenness centrality).


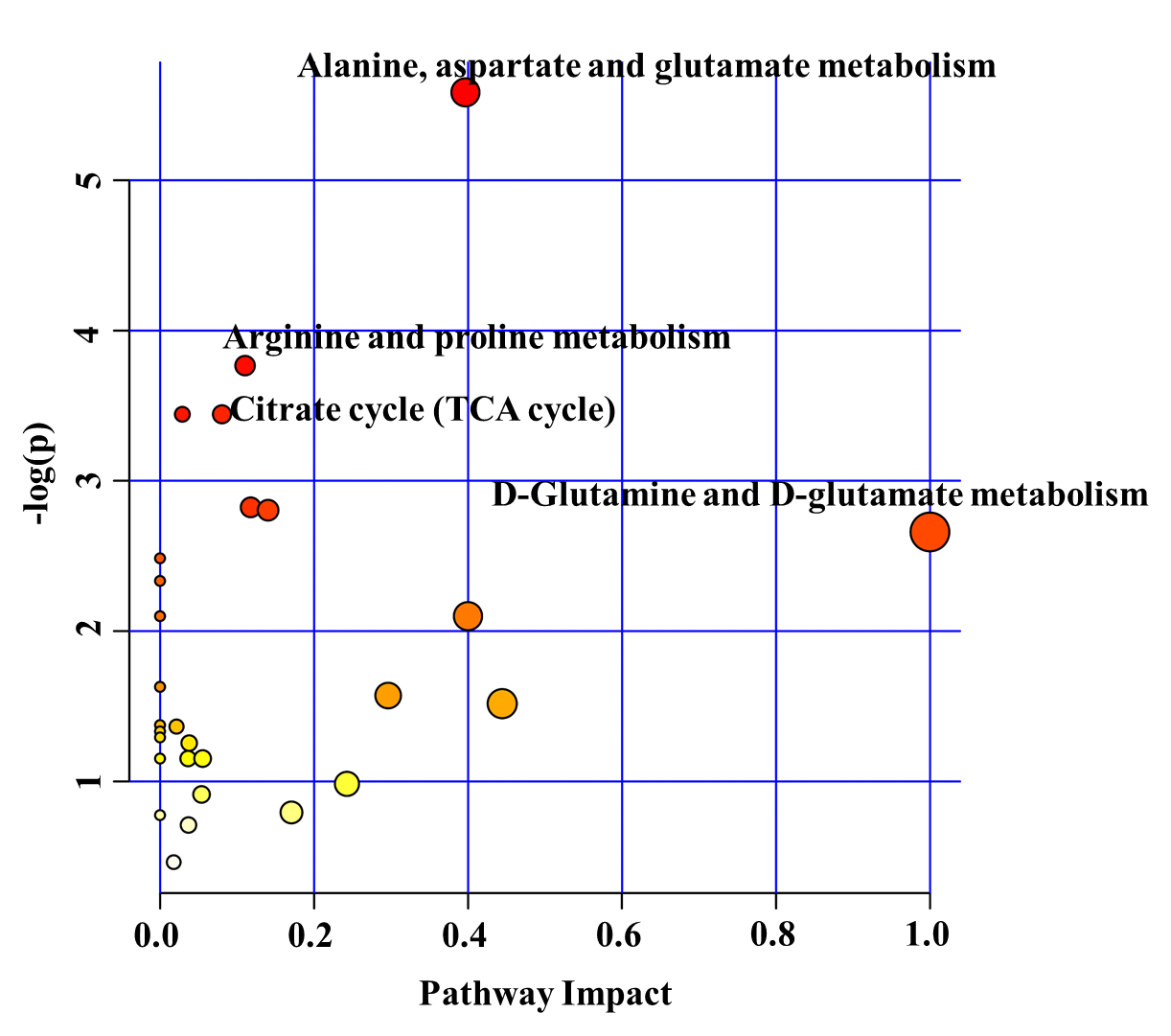


Fig. S3. Receiver operating characteristic (ROC) analysis of the biomarker panels suggested by binary logistic regression analysis

| Model | AUC Value | 95% CI |
| --- | --- | --- |
| Malic acid | 0.863 | 0.750-0.938 |
| Malic acid  Methionine | 0.945 | 0.854-0.987 |
| Malic acid  Methionine  Tryptophan | 0.983 | 0.910-1.000 |
| Glucose  Malic acid  Methionine  Tryptophan | 0.991 | 0.924-1.000 |
| GABA  Malic acid  Glucose  Methionine  Tryptophan | 1 | 0.940-1.000 |

Fig. S4. Comparison of discrimination power among biomarker, Bilirubin, and PT based on receiver operating characteristic (ROC) analysis

|  | Biomarker | Bilirubin | PT | Biomarker  +  Bilirubin | PT  +  bilirubin |
| --- | --- | --- | --- | --- | --- |
| 14h | 0.989  SN:100.00 SP:92.31 | 0.692  SN:71.43 SP:61.54 | 0.956  SN:85.71 SP:82.31 | 1.000  SN:100.00 SP:100.00 | 0.956  SN:95.71 SP:92.31 |
| 30h | 0.978  SN:100.00 SP:92.31 | 0.890  SN:100.00 SP:84.62 | 1.000  SN:100.00 SP:100.00 | 1.000  SN:100.00 SP:100.00 | 1  SN:100.00 SP:100.00 |
| 48h | 1.000  SN:100.00 SP:100.00 | 0.923  SN:100.00 SP:84.62 | 1.000  SN:100.00 SP:100.00 | 1.000  SN:100.00 SP:100.00 | 1  SN:100.00 SP:100.00 |
| All time | 0.993  SN:100.00 SP:94.87 | 0.805  SN:66.67 SP:84.62 | 0.976  SN:100.00 SP:84.62 | 1.000  SN:100.00 SP:100.00 | 0.979  SN:100.00 SP:84.62 |

SN, sensitivity SP, specificity
